# Implementation and evaluation of a Project ECHO telementoring program for the Namibian HIV workforce

**DOI:** 10.1186/s12960-020-00503-w

**Published:** 2020-09-01

**Authors:** Leonard Bikinesi, Gillian O’Bryan, Clay Roscoe, Tadesse Mekonen, Naemi Shoopala, Assegid T. Mengistu, Souleymane Sawadogo, Simon Agolory, Gram Mutandi, Valerie Garises, Rituparna Pati, Laura Tison, Ledor Igboh, Carla Johnson, Evelyn M. Rodriguez, Tedd Ellerbrock, Heather Menzies, Andrew L. Baughman, Laura Brandt, Norbert Forster, John Scott, Brian Wood, Kenton T. Unruh, Sanjeev Arora, Michelle Iandiorio, Summers Kalishman, Sarah Zalud-Cerrato, Jutta Lehmer, Stephen Lee, Mohammed A. Mahdi, Samantha Spedoske, Alexandra Zuber, Brigg Reilley, Christian B. Ramers, Ndapewa Hamunime, Gabrielle O’Malley, Bruce Struminger

**Affiliations:** 1 Directorate of Special Programmes, Namibian Ministry of Health and Social Services (MoHSS), Ministerial Building Harvey Street, Windhoek, Namibia; 2 International Training and Education Center for Health (I-TECH), University of Washington, 908 Jefferson Street, Seattle, WA USA; 3 US Centers for Disease Control and Prevention (CDC) Windhoek, Namibia, Florence Nightingale Street, Windhoek, Namibia; 4 Avacare Health, 8 Skietlood Street, Isando, South Africa; 5 US Centers for Disease Control and Prevention (CDC), Lusaka, Zambia, Independence Avenue, Lusaka, Zambia; 6 Namibian National Institute of Pathology (NIP), Ooievaar Street, Windhoek, Namibia; 7 US Centers for Disease Control and Prevention (CDC), 1600 Clifton Road, Atlanta, GA USA; 8 International Training and Education Center for Health (I-TECH), 4 Storch Street, Windhoek, Namibia; 9 Departments of Medicine, Division of Allergy and Infectious Diseases, University of Washington, 410 9th Avenue, Seattle, WA USA; 10 Mountain West AIDS Education and Training Centre (MWAETC), University of Washington, 908 Jefferson Street, Seattle, WA USA; 11 ECHO Institute, University of New Mexico, 1650 University Boulevard NE, Albuquerque, NM USA; 12 Elizabeth Glaser Pediatric AIDS Foundation (EGPAF), 1140 Connecticut Avenue NW, Washington, DC USA; 13 Ata Health Strategies LLC, 55 M Street NE #1012, Washington, DC USA; 14 NW Tribal Health Board (NPAIHB), 2121 SW Broadway STE 300, Portland, OR USA; 15 Family Health Centers of San Diego, University of San Diego School of Medicine, 823 Gateway Center Way, San Diego, CA USA

**Keywords:** HIV, Project ECHO, Community of Practice, Namibia

## Abstract

**Background:**

The Namibian Ministry of Health and Social Services (MoHSS) piloted the first HIV Project ECHO (Extension for Community Health Outcomes) in Africa at 10 clinical sites between 2015 and 2016. Goals of Project ECHO implementation included strengthening clinical capacity, improving professional satisfaction, and reducing isolation while addressing HIV service challenges during decentralization of antiretroviral therapy.

**Methods:**

MoHSS conducted a mixed-methods evaluation to assess the pilot. Methods included pre/post program assessments of healthcare worker knowledge, self-efficacy, and professional satisfaction; assessment of continuing professional development (CPD) credit acquisition; and focus group discussions and in-depth interviews. Analysis compared the differences between pre/post scores descriptively. Qualitative transcripts were analyzed to extract themes and representative quotes.

**Results:**

Knowledge of clinical HIV improved 17.8% overall (95% confidence interval 12.2–23.5%) and 22.3% (95% confidence interval 13.2–31.5%) for nurses. Professional satisfaction increased 30 percentage points. Most participants experienced reduced professional isolation (66%) and improved CPD credit access (57%). Qualitative findings reinforced quantitative results. Following the pilot, the Namibia MoHSS Project ECHO expanded to over 40 clinical sites by May 2019 serving more than 140 000 people living with HIV.

**Conclusions:**

Similar to other Project ECHO evaluation results in the United States of America, Namibia’s Project ECHO led to the development of ongoing virtual communities of practice. The evaluation demonstrated the ability of the Namibia HIV Project ECHO to improve healthcare worker knowledge and satisfaction and decrease professional isolation.

## Background

The Namibian Ministry of Health and Social Services (MoHSS), in collaboration with the US President’s Emergency Plan for AIDS Relief (PEPFAR) Namibia, implemented an acceleration plan to rapidly scale-up HIV care and treatment interventions starting in 2015 [1]. Since then, the MoHSS, with PEPFAR support, has further decentralized antiretroviral therapy (ART) services and expanded non-physician healthcare worker capacity to provide HIV services [1]. For Namibian HIV healthcare workers, clinical mentorship and in-service training are essential methods for building competencies and reinforcing skills to deliver high-quality clinical services [2]. Persistent staff shortages, traditional trainings conducted away from clinical duty stations, and low rates of healthcare worker retention, however, challenge effective HIV service provision to the estimated 14% (230 000 individuals) of the population living with HIV [1, 3, 4]. Additionally, Namibia is a large country with a small population (2.4 million), and communities are separated by long distances, which can result in healthcare workers feeling socially and professionally isolated [5]. Namibia needs innovative interventions that expand clinical capacity, increase professional satisfaction and retention, and strengthen decentralization and task sharing to expand HIV clinical services to achieve HIV epidemic control [1]. Continuing medical education delivered through virtual strategies including telehealth, telementoring, and vertically integrated learning such as virtual communities of practice have been shown to facilitate peer communication and knowledge sharing, reduce isolation, and increase intention to continue work in remote areas in both low- and high-resource settings [6, 7].

Project ECHO (Extension for Community Healthcare Outcomes) supports case-based community of practice education and training sessions through multipoint video conferencing [8]. The Project ECHO model connects multidisciplinary subject matter experts at a central hub with local healthcare workers at distant spoke sites through a combination of weekly didactic sessions and clinical case presentations and discussion. Following the Project ECHO model’s initial success increasing healthcare workers’ capacity to treat hepatitis C in underserved communities in the United States of America, more than 35 countries have replicated the model to improve services for various complex medical conditions [8, 9]. The Namibian MoHSS piloted the first HIV Project ECHO initiative in Africa at 10 Namibian clinical sites that provide care for more than 60 000 people living with HIV between November 2015 and September 2016; it evaluated the pilot in late 2016. This article describes the evaluation results and the resulting ECHO expansion.

## Methods

In 2014–2015, the MoHSS led an international consortium of partners to adapt the Project ECHO model for Namibia. The consortium developed a 9-month curriculum, engaged local partners to ensure adequate Internet access (1 mbps or better) for video conferencing, and secured Continuing Professional Development (CPD) accreditation with the local Health Professions Councils. The curriculum was developed in consultation with providers, mentors, and technical experts and covered clinical subject areas including opportunistic infections, antiretroviral therapy, prevention of mother to child transmission, tuberculosis co-infection, counseling discordant couples, and pediatric disclosure.

**Fig. 1:**
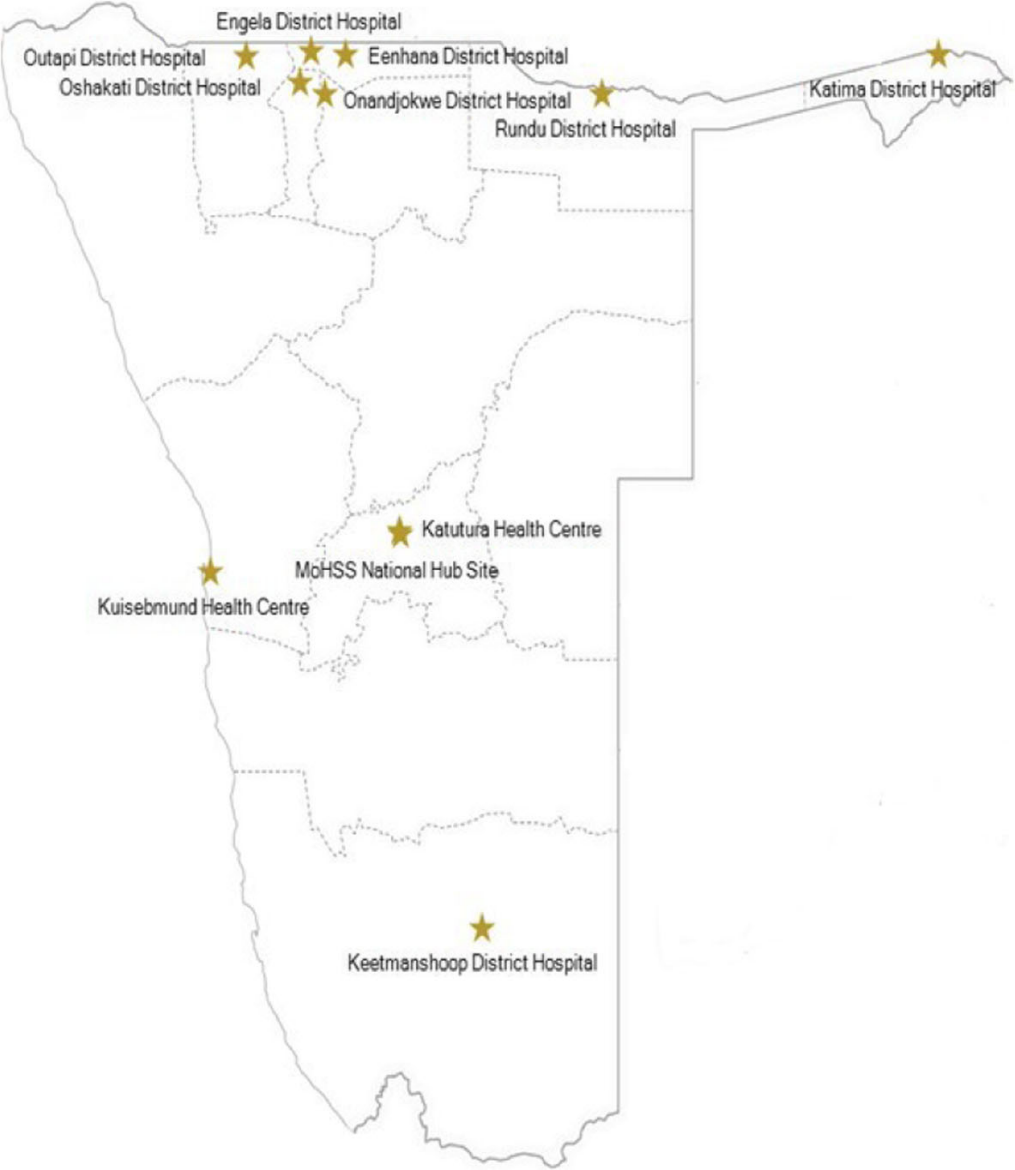
Map of Namibia HIV ECHO pilot sites

The MoHSS selected 10 pilot sites in regions with high HIV prevalence and reliable Internet connectivity per a site connectivity assessment (Fig. 1). The MoHSS chief physician clinical mentor, chief nurse mentor, and a Project ECHO program administrator/information technology specialist hosted and facilitated weekly sessions (excluding holidays) averaging 90-min in length via the Zoom video-conferencing platform from the hub in Windhoek, Namibia [10]. Sessions were held in the late afternoon to increase provider ability to participate as patient volumes are typically higher in the morning in public health facilities throughout Namibia.

Rotating subject matter experts from the MoHSS and other local and international academic and public health institutions supported the program by providing expert consultation. Spoke site participants included regional nurse and physician clinical mentors, physicians, nurses, pharmacists, laboratorians, health assistants (HIV counseling and testing cadre), and other members of the MoHSS healthcare team at formal pilot sites. The hub team tracked weekly participation to monitor participant interest and engagement and ensure CPD accreditation. As a complement to the Project ECHO model, the MoHSS national clinical mentorship team provided critical leadership and technical support to the regional mentors and spoke sites. Regional clinical mentors served as community site leaders, helping facilitate ECHO didactic sessions, preparing case presentations, and encouraging participation and information sharing across sites.

Physicians, nurses, and pharmacists at pilot sites who participated in at least two Project ECHO sessions were eligible for inclusion in the evaluation. All individuals participating in the Project ECHO sessions provided verbal informed consent. Individuals who participated in the focus group discussions and in-depth interviews provided an additional verbal informed consent. The protocol was approved by the Namibia MoHSS and reviewed in accordance with the Centers for Disease Control and Prevention (CDC) human research protection procedures and was determined to be a nonresearch, program evaluation.

### Quantitative methods

The program implementation consortium conducted a mixed-methods evaluation to assess pilot program feasibility and acceptability, provider knowledge, provider satisfaction with Project ECHO, and professional satisfaction. Evaluation methods included pre/post program assessment of clinical knowledge, self-efficacy, and professional satisfaction; assessment of CPD credit acquisition; and focus group discussions and in-depth interviews. The Namibia Project ECHO consortium developed the 25-question clinical knowledge assessment using the MoHSS core competencies for clinical providers (physicians and nurses), the fifth edition of the Namibian National HIV Treatment Guidelines, input from the international Project ECHO consortium, and a rapid needs assessment. The same paper-based assessment was conducted at two points in time: once just prior to the first Project ECHO session (pre-assessment) and again at the completion of the pilot (post-assessment). Self-assessment of efficacy asked participants to rate their HIV knowledge, skills, and competence on a Likert scale ranging from “none or no skills” to “expert, able to teach others” in clinical subject areas covered in the Project ECHO curriculum. Participants also completed an assessment evaluating their perceived efficacy in several aspects of clinical program quality management (including the ability to determine quality gap causes and design and implement a quality improvement plan) and their overall professional satisfaction before and after the pilot period.

For each participant, we calculated the difference between pre/post program knowledge assessment scores. The mean difference between pre- and post-program test scores, along with the percent improvement from the pre-program mean test score and its 95% confidence interval (CI), was evaluated by profession and earned CPD credits. Knowledge assessment scores were not evaluated by additional characteristics due to the small number of participants observed for potential subgroups (e.g., Project ECHO site, participation in focus group discussion, in-depth interviews). We compared other pre/post program assessment components (i.e., self-efficacy, professional satisfaction, CPD credits) using descriptive statistics.

### Qualitative methods

The qualitative component of the evaluation used focus group discussions and in-depth interviews to assess the perceived usefulness of the Project ECHO sessions, participants’ ability to integrate learning into clinical practice, and acceptability and feasibility of the Project ECHO model. Interviews and focus group discussions occurred approximately 6 months into the 9-month pilot after approximately 25 Project ECHO sessions were held. We purposively sampled participants having attended at least two Project ECHO sessions by provider type and for geographic variability, as well as suggestions from regional clinical mentors. Participation was voluntary, and availability and willingness to participate were factors in participant selection. The program administrator/information technology specialist invited healthcare workers selected to participate via email or phone or during the Project ECHO sessions. Two individuals, one from CDC Namibia and one from the ECHO Institute at the University of New Mexico, conducted focus group discussions and in-depth interviews using the Zoom videoconferencing platform. All participants joined the interview and focus groups from their remote locations. The facilitators participated from the Namibia MoHSS boardroom where the weekly Project ECHO pilot sessions were held.

Each of the focus group discussions lasted approximately 1 h, while the interviews lasted approximately 30 min. An interview guide containing questions with sub-questions was developed and approved by the MoHSS. Based on the results of the first focus group discussion, however, several questions were added to obtain a more robust picture of how Project ECHO contributed to learning and elicit challenges and barriers to participation. Audio files from the focus group discussions and interviews were recorded using the Zoom videoconferencing platform and stored securely on password-protected MoHSS computers on the secure MoHSS network. Audio files were transcribed by a local professional transcriptionist without names or other unique identifiers.

Following an initial review of the de-identified and transcribed audio recordings, two reviewers from the International Training and Education Center for Health (I-TECH) at the University of Washington independently developed themes and codes using an inductive approach, discussed discrepancies, and finalized a codebook. Both reviewers coded a single transcript and compared results to validate the finalized codebook. A single coder used the codebook to analyze and extract themes, sub-themes, and representative quotes from transcripts. The second reviewer randomly selected coded samples for secondary review in addition to reviewing and revising final themes, sub-themes, and representative quotes. Evaluation stakeholders reviewed and provided feedback on a qualitative summary report to add context to themes and recommendations.

## Results

### Quantitative analysis

During the pilot (November 2015–September 2016), the program-implementing consortium conducted 34 Project ECHO sessions with each pilot site attending between 18 and 33 sessions. Each session had an average of 79 participants (range 44–133) and one case presentation (range 0–3).

One-hundred and fifty-five participants took the pre knowledge assessment, and 78 participants from nine clinical sites (one clinical site did not submit post assessment scores for analysis) took both the pre and post clinical program-knowledge assessments. The average age of participants was 38.9 years, and 63.1% of participants were female. The average length of professional experience was 5.7 years (range 0–15 years), and on average, it was reported, each provider was caring for 263 HIV patients per week. Most participants were nurses (53.8%), who also demonstrated the largest increase in knowledge (11.7 percentage points; 22.3% (95% CI 13.2–31.5%) improvement). Physicians demonstrated the least improvement (2.2 percentage points) but had the highest pre/post program knowledge assessment scores (Table 1). Cadres included in the “Others” category were administrative officers, social workers, data clerks, and an HIV testing quality assurance officer.

**Table 1:** Evaluation of knowledge assessment scores before and after a telementoring intervention for HIV healthcare workers in Namibia, by profession and Continuing Professional Development (CPD) credits

				Difference between pre- and post-program test scores	
Characteristic	Number of participants (%)	Pre-program mean test score	Post-program mean test score	Mean	Percent improvement from pre-program mean test score (95% CI)
Profession					
All	78 (100.0)	54.5	64.2	9.7	17.8 (12.2–23.5)
Physician	11 (14.1)	72.7	74.9	2.2	3.0 (− 4.3 to 10.3)
Nurse	42 (53.8)	52.4	64.1	11.7	22.3 (13.2–31.5)
Others	16 (20.5)	45.8	57.2	11.5	25.1 (10.6–39.7)
Pharmacist	9 (11.5)	57.3	64.0	6.7	11.7 (− 1.5 to 24.8)
Number of CPD credits					
Did not report	13 (16.7)	55.7	63.7	8.0	14.4 (− 0.6 to 29.3)
1–5 CPD credits	32 (41.0)	51.6	60.9	9.2	17.8 (9.5–26.3)
6–28 CPD credits	33 (42.3)	56.7	67.6	10.9	19.2 (9.6–28.8)
Profession and number of CPD credits*					
Physician					
1–5	6 (9.2)	70.0	75.3	5.3	7.6 (0.4–14.9)
6–28	3 (4.6)	80.0	84.0	4.0	5.0 (− 19.8 to 29.8)
Nurse					
1–5	14 (21.5)	47.4	57.1	9.7	20.5 (2.6–38.4)
6–28	21 (32.3)	54.3	67.4	13.1	24.1 (9.6–38.8)
Others					
1–5	6 (9.2)	36.7	48.7	12.0	32.7 (− 4.2 to 69.6)
6–28	7 (10.8)	53.7	63.4	9.7	18.1 (− 2.9 to 39.1)
Pharmacist					
1–5	6 (9.2)	58.0	67.3	9.3	16.0 (− 3.1 to 35.3)
6–28	2 (3.1)	58.0	60.0	2.0	3.4 (− 128.0 to 134.9)

*CI* confidence interval

*13 participants did not report CPD credits, reducing the total sample size in this group to 65

Analyses examined the association between knowledge assessment results and acquisition of CPD credits. For each 1-h Project ECHO session, participants earned one CPD credit. Categorization of CPD credit acquisition was based on the median number of participants earning credits between 1 (minimum) and 28 (maximum) credits: 1–5 credits earned (*n* = 32) and more than 5 credits earned (*n* = 33). The two groups had comparable gains in knowledge, with a mean increase of 9.2 percentage points for the group that earned 1–5 CPD credits and 10.9 percentage points for the group that earned 6–28 CPD credits.

We stratified pre/post program scores by profession and CPD groups to differentiate the effects of profession and number of CPD credits on knowledge improvement. The highest average difference in test scores occurred among nurses who attended > 5 sessions, with a significant increase of 13.1 percentage points, representing a 24.1% (95% CI 9.6–38.8%) improvement from the pre-assessment. All other mean differences in stratified profession and CPD groups were positive (range, 2.0–12.0 percentage points); these differences involved smaller sample sizes for CPD groups within professions (Table 1).

Analysis of the pre/post program self-efficacy and professional satisfaction assessments revealed that 66% of Project ECHO participants experienced reduced feelings of professional isolation. During the follow-up survey, 78% of the participants were satisfied or very satisfied with their general professional experience as compared to only 48% at baseline. Overall, there was improvement in participant self-efficacy in all domains assessed in the follow-up survey as compared to the baseline (range 17.4–40.9% difference from baseline). Participants also experienced improved understanding in HIV program quality management in the follow-up survey (5.9–16.9% difference from baseline). Moreover, 57% of participants reported that Project ECHO improved their access to CPD credits. Participants earned an average of 17.5 CPD credits through their participation in the 9-month pilot, meeting 58% of the annual requirement of 30 CPDs for registration with the Health Professions Councils.

### Qualitative analysis

Seven individuals participated in two focus group discussions, and seven additional individuals participated in single subject in-depth interviews. Participants represented 7 out of 10 pilot sites and included 10 nurses (71.4%), one pharmacist (7.2%), and three physicians (21.4%). Participants attended between two and 22 sessions.

Focus group discussions and in-depth interview participants described both case scenarios and didactic presentations as relevant to their clinical practice. Commonly recounted motivators for attending sessions included opportunities for peer-to-peer learning of evidence-based best practices from colleagues at other facilities.

The ones I have participated in are set up because of the few challenges we were meeting with some of the management of the patients. So we wanted to get more information from other sites, and help, on how they [are] dealing with some of those cases. That was the main motivating factor for attending some of the sessions. (Physician)

Non-physicians highlighted how attending Project ECHO sessions increased their confidence to provide HIV management and promoted HIV task sharing from physicians to nurses. For example, one participant shared that they previously referred all patients with high viral load results to a physician for intensive adherence counseling. Following an ECHO session, the participant began provision of intensive adherence counseling for high viral load patients, potentially helping to mitigate a service delivery bottleneck.

Yeah, those case studies, they help us a lot. Like for example, my previous colleague from X said, some of the cases we were just referring to the doctors, but with the help and introduction of ECHO, we manage to do those cases ourselves instead of referring those patients to the doctors. For example, a person having [high] viral load, most of them we used to refer to the doctors and now we manage to intensely counsel… (Nurse)

In addition, participants described improved ability to identify aspects of HIV clinical care requiring escalation to the level of a physician.

They help us to manage the minor cases like those cases where we were supposed to refer to a doctor every time, now from [the] ECHO sessions we manage to deal with some cases. (Nurse)

Participants also noted the advantages of participation in a distance-learning platform as including expanded access to training, cost-savings related to less travel and off-site training, reduced service disruption, and improved continuity of care (Table 2).

…It helps that even the human resources at the clinic are not affected. Workshops usually make staff go and patients remain, in a way it actually helps reduce the movement of staff at the hospital but at the same time they will be learning. (Physician)

**Table 2:** Representative themes and quotes after a telementoring intervention for HIV healthcare workers in Namibia, by respondent type

Theme	Sub-theme	Respondent type	Representative quote
Clinical Relevance	Immediate application	Nurse Nurse Nurse	“What I have learned from the discussion I have put in practice every day because it will help me.” “We [who work together] discuss after the ECHO session the same day or the next day. We used to talk about things that we learned, and we see also where we can incorporate some of these things [presented in the ECHO sessions] into our own clinics. So it was just amongst us as a group working at the ART clinic.” “After the session we used to discuss and give examples that we have come across within our Centre with our patients.”
Improved Practice	Task sharing	Nurse Pharmacy Assistant	“The reason I attend these ECHO session [is] it helps me manage some of these minor cases which cannot be attended to/cannot send to doctor, but the major cases we send to doctors, it helps a lot.” “Now we can monitor the [patient’s] viral load by even giving health education to our patients … nurses can attend [to this] now, not only referring them to the doctors.”
	Peer Learning	Pharmacy Assistant Physician	“It [ECHO] is very important, you are getting a lot of ideas from each other, you are sharing ideas, and you are also knowing what you do not know from others.” “The other thing about participating, it is ...about networking also, to help us to know who is doing what…so if I have a problem I know who to consult, who to talk to or refer the patient with.”
Training type	Distance learning	Physician Pharmacy Assistant Nurse	“I think it is a very good program especially for us who are a bit far from the capital city where we cannot attend all of the trainings.” “…With a workshop, it is only one or two people who can attend from the whole facility, but with ECHO, a lot can attend and can have also [share] ideas. One person can go to the workshop and he/she might not give clear feedback from what he/she really got from the workshop.” “ECHO is good because it saves money [as opposed to] workshops where people [are] supposed to travel long distances and book accommodations. But with ECHO you are at your working place, you take your available time to attend and then you get the information.”

Participants had several suggestions for how the program might be improved to better accommodate HIV provider time constraints. For some participants, participation during clinic hours was challenging due to high patient loads/or volumes and because of broadband limitations. One nurse reported:

Unfortunately I could not attend most of them [ECHO sessions] due to network problems at our side… and also maybe the facility due to shortage of staff. (Nurse)

Participants suggested Project ECHO adapt a more flexible schedule to accommodate each site’s high volume patient times and making recorded sessions available to participants unable to attend live.

Participants also suggested extended participation, session content, and language be further tailored to nurses and additional healthcare worker cadres, such as field promoters and HIV counselors and testers who are the cadres with whom patients have significant contact, but for whom some of the content may be too advanced.

So the ECHO Program caters [to] all people, the Counselors, the Nurses and the Doctors, so when you compare the level of knowledge about the guidelines [between them] it is different. So there are some topics which you can attend but you think this is too simple for me, like you already know everything. But for the nurses it will be new for them. But sometimes if there could be scenarios for accommodating the nurses and other staff, like the counselors. (Physician)

Finally, program participants also suggested increasing the ownership, accountability, and participation of non-physician healthcare workers by having them develop session topics and presenting didactic lectures and cases.

## Discussion

In Namibia, Project ECHO developed a robust virtual community of practice and learning. Participants demonstrated increased knowledge, reported increased capacity and improvements in their clinical practice, expressed reduced feelings of professional isolation, and cited promotion of peer-to-peer cross-facility learning as an important motivator for attending sessions.

### Increased capacity

Our evaluation showed that healthcare worker knowledge about clinical management of HIV increased after the Project ECHO sessions, with nurses having the most significant knowledge increase, and that increases in knowledge led to increased feelings of self-efficacy. Self-efficacy, an individual’s confidence in his or her ability to accomplish specific work tasks, plays an important role in determining an individual’s interest and persistence in performing a particular work task [11, 12]. In the current context of decentralizing HIV care in Namibia, nurses provide most HIV care and treatment services. Nurses described both increased self-efficacy and were able to specify in qualitative findings how those feelings translated into improved clinical service delivery through task sharing.

Our findings of increased HIV clinical knowledge and self-efficacy through Project ECHO implementation in sub-Saharan Africa are similar to evaluations done in high-resource settings, such as the United States of America. An evaluation of a clinical HIV Project ECHO program in the United States of America found significant increases in provider confidence to manage many aspects of HIV care and reduced feelings of professional isolation [13]. Project ECHO evaluations in fields other than HIV have found similar results. A study of a mental health and addictions Project ECHO intervention in India identified significant increases in learning and self-confidence [14]. An evaluation of a Project ECHO program for chronic pain in the United States of America found improved self-confidence and knowledge for participating providers, as did an assessment of the hepatitis C Project ECHO in New Mexico, which found improved self-efficacy, knowledge, and professional satisfaction [15, 16].

### Professional isolation

Our evaluation also showed that engagement in Project ECHO sessions reduced feelings of professional isolation, a result which has also been found in evaluations of ECHO implementation in rural and underserved communities in the United States of America [16]. In sub-Saharan Africa, professional isolation has been reported as a key component of job dissatisfaction [11, 17, 18]. In sub-Saharan Africa, lack of professional development opportunities has been cited as a key non-financial factor contributing to decreased worker motivation which negatively impacts health service delivery [19]. In addition, poor retention of healthcare workers in rural areas in sub-Saharan Africa is especially problematic, leaving large segments of the population without access to trained health care providers [20]. Evidence from a six-country study (Cameroon, Ghana, Senegal, South Africa, Zimbabwe) found one of the main reasons for departure from public health service positions was the desire for professional education [21]. Increased connectedness with other health professionals is also important for nurturing a professional identity and shared cultural norms [11].

### Peer learning

The sociocultural perspective of learning as a collaborative co-construction of knowledge provides the theoretical foundation for learning in the Project ECHO model [22]. Situated learning theory emphasizes that through collaboration with more knowledgeable peers, an individual can perform at a higher level of complexity than he or she can do alone [22]. Collaboration, encouragement, and sharing among those working in similar contexts with varying levels of knowledge and experience are key to achieving higher-level tasks of increasing complexity [22]. Online learning supplemented with structured peer coaching program has been shown to significantly improve the successful acquisition of technical proficiency of new surgical skills, in comparison to learners who only learned from an online learning resource [23]. The collective potential of a group can only be realized if each member is aware of the knowledge of others and can capitalize on it by offering and receiving help through co-mentoring, which the Project ECHO model amplifies [24].

Though peer-to-peer learning is meant to be in-depth and frequent, it can be challenging to deliver on a large scale [25]. Project ECHO is designed to mitigate these challenges and can promote large-scale delivery of peer-to-peer learning [16]. The model’s case-based reasoning approach encourages collaborative problem solving to accelerate individual learning processes and assimilate and actualize content [16]. Facilitation and scale-up of collaborative learning through peer networks, including through implementation of Project ECHO, has positive implications for more efficient diffusion of evidence-based healthcare information [24, 26].

### Virtual communities of practice

Participants in our study reported that one of the key components of the Project ECHO platform is the creation of virtual communities of practice. Communities of practice are described as, “groups of people who share a concern or passion for something they do and learning how to do it better as they interact regularly” [27]. Studies of virtual communities of practice in other contexts demonstrate the integral role they play in interprofessional learning and collaboration by providing opportunities to overcome challenges linked to in-person interaction such as linking geographically dispersed networks of people with common interests [28–32].

Active participation and engagement, however, are integral to ensure sustainability of virtual communities of practice. Active participation is fostered by having supportive leadership including local champions and active facilitation [28, 29]. Facilitation and active support were provided in Namibia through integration with the existing regional clinical mentorship program. Regional, onsite clinical mentors encouraged Project ECHO participation, assisted with participant case-presentation development, and often continued discussions with participants after sessions concluded, increasing engagement with the content. The degree to which an innovative approach or intervention can be incorporated into existing organizations or systems is an important factor in its sustainable implementation [33].

### Project ECHO expansion

The Namibia HIV Project ECHO unofficially expanded via word of mouth, with healthcare workers from six additional sites not belonging to one of the 10 pilot sites joining during the pilot period. Following the pilot and review of the positive evaluation results, MoHSS formally launched Project ECHO in late 2017 and, as of May 2019, has expanded the program to a total of 40 clinical spoke sites that provide care to more than 140 000 people living with HIV. Project ECHO is also being adapted to address specific populations and topic areas within HIV program services, such as supporting healthcare workers in the implementation of community adherence clubs for stable patients to improve retention. MoHSS is also considering expanding implementation of Project ECHO for programmatic areas beyond HIV (e.g., multi-drug resistant tuberculosis, laboratory services) and routinely uses the Zoom videoconferencing platform for a variety of educational and program management activities, representing catalytic ripple effects of the Project ECHO program. Following the success in Namibia, implementation of the Project ECHO model has expanded rapidly across Africa. Currently, there are over 30 Project ECHO programs related to HIV, tuberculosis, laboratory systems strengthening, and global health security being implemented in over 14 countries in sub-Saharan Africa, along with several multi-country Project ECHO programs that engage more than 40 countries across sub-Saharan Africa [34–37].

### Limitations

This evaluation is subject to several limitations. First, while the evaluation demonstrated an increase in provider knowledge and self-efficacy, it did not assess impact on patient outcomes. The Project ECHO model, however, has demonstrated results in improved patient outcomes in other settings and disease areas [38, 39]. Second, though participants experienced increased satisfaction and decreased feelings of isolation, this evaluation did not assess provider retention. Third, focus group discussions and in-depth interviews were limited to only 14 individuals, so these findings may not be generalizable. In addition, assessment of clinical knowledge was limited by small sample sizes in several participant subgroups. Lastly, the evaluation was unable to differentiate increases in provider knowledge, satisfaction, and self-efficacy directly related to the HIV Project ECHO pilot program from the MoHSS clinical mentorship program, which was implemented concurrently.

## Conclusions

The success of the Namibia HIV Project ECHO pilot demonstrated the feasibility of adapting the Project ECHO model to a sub-Saharan African setting. In Namibia, the Project ECHO model has successfully promoted distance learning through virtual communities of practice, while reducing professional isolation and increasing self-efficacy in healthcare workers. By helping achieve the goal of delivering the right knowledge to the right place at the right time for those who need it most, the Project ECHO model has proven to be a powerful tool for helping achieve successful decentralization of HIV clinical services across Namibia [40].

## Data Availability

The datasets used and/or analyzed during the current study are available from the corresponding author on reasonable request.
